# Downregulation of *EHT1* and *EEB1* in *Saccharomyces cerevisiae* Alters the Ester Profile of Wine during Fermentation

**DOI:** 10.4014/jmb.2201.01008

**Published:** 2022-04-10

**Authors:** Xue Yang, Xuenan Zhang, Xi He, Canzhen Liu, Xinjie Zhao, Ning Han

**Affiliations:** 1 Shandong Provincial Key Laboratory of Microbial Engineering, School of Biologic Engineering, Qilu University of Technology (Shandong Academy of Sciences), Jinan 250300, P.R. China; 2 State Key Laboratory of Biobased Material and Green Papermaking, Qilu University of Technology (Shandong Academy of Sciences), Jinan 250300, P.R. China

**Keywords:** Fermentation esters, diploid wine yeast EC1118, *EEB1*, *EHT1*, decrease of gene expression level

## Abstract

*EHT1* and *EEB1* are the key *Saccharomyces cerevisiae* genes involved in the synthesis of ethyl esters during wine fermentation. We constructed single (*Δeht1*, *Δeeb1*) and double (*Δeht1Δeeb1*) heterogenous mutant strains of the industrial diploid wine yeast EC1118 by disrupting one allele of *EHT1* and/or *EEB1*. In addition, the aromatic profile of wine produced during fermentation of simulated grape juice by these mutant strains was also analyzed. The expression levels of *EHT1* and/or *EEB1* in the relevant mutants were less than 50% of the wild-type strain when grown in YPD medium and simulated grape juice medium. Compared to the wild-type strain, all mutants produced lower amounts of ethyl esters in the fermented grape juice and also resulted in distinct ethyl ester profiles. *ATF2*, a gene involved in acetate ester synthesis, was expressed at higher levels in the *EEB1* downregulation mutants compared to the wild-type and *Δeht1* strains during fermentation, which was consistent with the content of acetate esters. In addition, the production of higher alcohols was also markedly affected by the decrease in *EEB1* levels. Compared to *EHT1*, *EEB1* downregulation had a greater impact on the production of acetate esters and higher alcohols, suggesting that controlling *EEB1* expression could be an effective means to regulate the content of these aromatic metabolites in wine. Taken together, the synthesis of ethyl esters can be decreased by deleting one allele of *EHT1* and *EEB1* in the diploid EC1118 strain, which may modify the ester profile of wine more subtly compared to the complete deletion of target genes.

## Introduction

Several volatile compounds, including alcohols, esters, acids, aldehydes and ketones, are produced during the fermentation of grape juice in winemaking, and affect the aroma and sensory quality of wine [1]. Most of these aromatic substances are produced as a result of the metabolic breakdown of sugars by *Saccharomyces cerevisiae* [2, 3]. Ethyl esters of fatty acids are also produced by the yeast cells during alcoholic fermentation, and have a significant impact on the sensory quality of wine with a lower threshold [4]. Medium-chain fatty acid (MCFA) ethyl esters such as ethyl butyrate, ethyl caproate, ethyl octanoate and ethyl decanoate [5, 6] are formed via the condensation of ethanol and fatty acid acyl-CoA or fusel acyl-CoA by ethanol-O-acyltransferases [7, 8]. Fatty acid acyl-CoA is produced during the synthesis of fatty acids, whereas fusel acyl-CoA is a product of carbon and nitrogen sources [9]. The key enzymes involved in the synthesis of MCFA ethyl esters are Eht1p (ethanol hexanoyl transferase 1) and Eeb1p (ethyl ester biosynthesis 1), which are respectively encoded by *EHT1* and *EEB1* [10].

A previous study showed that *EHT1* and *EEB1* knockout strains of yeast produce significantly lower amounts of ethyl ester, although Eht1p had less impact on the synthesis of MCFA ethyl esters compared to Eeb1p [10]. Consistent with this, the ethyl ester content increased significantly in wine fermented with an *EHT1*-overexpressing industrial wine yeast strain (VIN13) compared to that with the standard strain [11]. Zhuang *et al*.[12] also reported a considerable increase in the content of ethyl esters in the basic wort medium fermented with *Pichia pastoris* strains overexpressing *EHT1* or *EEB1*. However, the *EHT1* and *EEB1* overexpression mutants of the BY4741and CMBS SS01 strains do not produce higher levels of MCFA ethyl esters during fermentation compared to the standard strains due to the bifunction of the synthesis and hydrolysis functions of both enzymes [10, 13]. This discrepancy can be ascribed to the different genetic backgrounds of the host strains [11].

Higher monohydric alcohols containing three carbon atoms [14] are formed as a result of the decarboxylation and reduction of α-keto acids, which in turn are derived from glycolysis or transamination of amino acids [9, 15]. While appropriate amounts of esters formed during the fermentation of higher alcohols enhance the complexity of wine aroma, superfluous higher alcohols have a negative impact on the taste [16]. For instance, acetate esters are frequently produced during alcoholic fermentation and impart fruity notes [5, 17]. They are synthesized through acetyl transfer from acetyl-CoA to alcohol by the acetyltransferases ATF1 and *ATF2* [4, 18], and the deletion and overexpression of *ATFs* only affect acetate ester synthesis and not that of ethyl esters [4, 19]. The interaction of MCFA ethyl esters and the acetate ester synthesis pathway remains unclear.

The genetic background of the yeast strains, fermentation substrate and environmental conditions markedly influence the final aroma profile of wine. Most studies have used haploid yeast strains to analyze the impact of their genes on fermentation and wine characteristics. However, the industrial strains of yeast are typically diploid, and produce esters and other flavor compounds more efficiently than laboratory haploid strains [11, 13]. Therefore, we generated *EHT1* or/and *EEB1* deletion mutants of the industrial wine yeast EC1118, one of the most widely used strains for wine production globally, through homologous recombination. The wild-type and mutant strains were then used to ferment simulated grape juice to assess the influence of *EHT1* and *EEB1* on the production of esters during fermentation.

## Materials and Methods

### Construction of *EHT1* and *EEB1* Deletion Cassettes

Plasmid DNA was prepared using a FastPure Plasmid Mini Kit (Vazyme Biotech, China) and High Fidelity DNA polymerase (Vazyme Biotech) was used for PCR. The Cre-loxP system was used to inactivate one allele of the target locus with the kanMX marker [21, 22]. The *loxP-kanMX-loxP* fragment was amplified from the pUG6 plasmid using the loxP-KanMXloxP-F1 and *loxP-KanMX-loxP*-R1 (*EHT1*) or the *loxP-KanMX-loxP*-F2 and *loxP-KanMX-loxP*-R2 (*EEB1*) primers. To construct the deletion cassette for *EHT1* (EA1KEB1), its 5’ and 3’ flanking regions were amplified using the EA1-F/EA1-R and EB1-F/EB1-R primer pairs respectively and fused with *loxP-kanMX-loxP* by homologous recombination using the ClonExpress Entry One Step Cloning Kit (Vazyme Biotech). The EA2KEB2 deletion cassette was similarly constructed using the EA2-F/ EA2-R and EB2-F/ EB2-R primers that amplified the 5’ and 3’ flanking regions of *EEB1*, and the amplified fragment was fused with *loxP-kanMX-loxP*. The double deletion strain, *Δeht1Δeeb1*, was constructed from the *Δeeb1*strain by the above method. The deletion cassettes were verified by sequencing, and the primers used for cloning are listed in Table S1.

### Yeast Transformation and Screening

The deletion cassettes were transformed into the diploid EC1118 cells by the LiAc-PEG method [20], and the transformants were selected on YPD plates containing 400 μg/l G418 (Sigma) for 2-3 days at 30°C. The *Δeht1* mutants with the EA1KEB1 deletion cassette were verified by PCR using EA1-F and EB1-R primers, and the EA2-F and EB2-R primers were used to identify the *Δeeb1* strains with the EA1KEB2 deletion cassette. The double mutants were verified likewise. The mutant strains were then transformed with the Cre-expressing pSH65 plasmid, and Cre recombinase was induced on YP+2% galactose to remove the *kanMX* label.

### Microbial Strains, Plasmids, Media and Culture Conditions

The yeast and bacterial strains and plasmids used in this study are listed in Table S2. The yeast cells were inoculated into YPD medium (10 g/l yeast extract, 20 g/l peptone and 20 g/l glucose) and cultured at 30°C with constant shaking at 200 rpm. The galactose-containing yeast peptone glucose (YPG) medium was used to induce the expression of Cre enzyme. The kanamycin and zeocin-resistant transformants were screened using G418 (400 μg/l) and Zeocin (2,500 μg/l) (Solarbio, China), respectively. *Escherichia coli* DH5α was grown in Luria-Bertani (LB) medium (5 g/l yeast extract, 10 g/l tryptone and 10 g/l NaCl) at 37°C with constant shaking at 200 ×*g*. The clones expressing the gene deletion cassettes were selected on 1.5% agar media using kanamycin (50 μg/l) (Solarbio).

### Real-Time Quantitative PCR

Total RNA was extracted from the yeast cells using the Yeast RNAiso Kit (Takara, China), and transformed into cDNA using a reverse transcription kit (Vazyme Biotech). Quantitative PCR (qPCR) was performed on a real-time PCR system (Bio-Rad iQ5) using the SYBR Green Kit (Vazyme Biotech). The thermal cycling conditions were as follows: 95°C for 2 min followed by 40 cycles of 95°C for 15 s and 56°C for 10 s, and a melting cycle from 65°C to 95°C. The housekeeping gene *actin* was used as the internal control, and the transcript levels were quantified using the 2^−ΔCT^ formula, where ΔCT is the number of PCR cycles required for the log phase of amplification for the test gene minus that of *actin* [23]. The primer sequences are listed in Table S1.

### Growth Curve and Fermentation

The yeast cells were pre-cultured in 10 ml YPD medium at 30°C for 24 h. The OD_600_ was measured, and the cells were respectively inoculated into fresh YPD medium and chemically defined grape (CDG) medium at the density equivalent of 0.1 OD_600_. The cells inoculated in the YPD medium were cultured at 30°C and the cell density was measured every 2 h. The CDG medium was fermented at 25°C for 12 days, and the loss of CO_2_ was measured every 12 h. The concentrations of residual sugars, alcohol and total acids were determined at the end of the fermentation process.

### Gas Chromatography-Mass Spectrometry

The fermented samples were analyzed qualitatively and quantitatively by gas chromatography-mass spectrometry (GC-MS) as described previously with some modifications [24]. Briefly, each sample was subjected to a topical solid-phase microextraction method using a 50/30 μm DVB/CAR/PDMS extracted head, following which a 10-ml sample, 1 g NaCl and 30 μl internal standard 4-methyl-2-pentanol (2 g/l) were mixed in a bottle. After pre-heating at 40°C for 10 min, the samples were agitated at 500 rpm and extracted at the requisite temperature for 60 min, followed by high-temperature resolution for 8 min. The chromatographic column temperature program was as follows: 40°C for 8 min, steady increase till 70°C with increments of 5°C per minute, hold at 70°C for 3 min, steady increase till 230°C at the rate of 5°C per minute, and hold at 2 min. For EI ion bombardment, the electron energy was 70eV and the ion source temperature was 200°C on full-scan mode. The results were preliminarily retrieved and analyzed using the computer spectrum library (NIST14). The standard internal method was used for quantitative analysis.

### Statistical Analysis

All data were expressed as the mean ± standard deviation (SD) of three replicates. One-way analysis of variance (ANOVA) and LSD test (*p* < 0.05) were used to test for significant differences between the mean values. The SPSS19.0 software (SPSS Inc., USA) was used for all statistical analyses.

## Results and Discussion

### Relative Expression Levels of *EHT1* and *EEB1* in the EC1118 Mutants

**Fig. 1:**
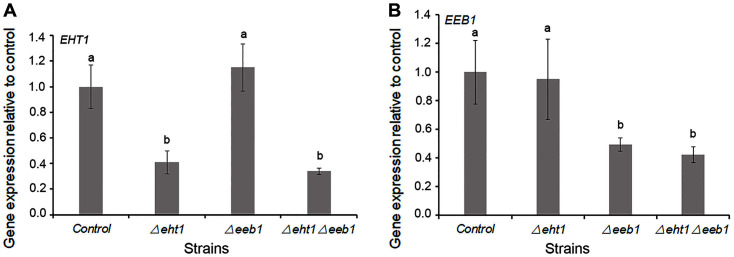
Relative expression levels of *EHT1* (**A**) and *EEB1* (**B**) in the yeast strains grown in YPD medium. Values are the mean ± SD of three experiments. Different letters (a-c) indicate significant differences between strains calculated by LSD test (*p* < 0.05).

We generated the *Δeht1*, *Δeeb1* and *Δeht1Δeeb1* mutant strains of the wine yeast EC1118 by replacing *EHT1* and *EEB1* genes with the homologous deletion cassettes EA1KEB1 and EA2KEB2, respectively. Given that EC1118 is a diploid strain and contains two alleles of *EHT1*and *EEB1*, the mutant strains were obtained by disrupting one allele of each target locus. To assess the effect of gene deletion, the relative expression levels of *EHT1* and *EEB1* were detected in the mutant strains grown in YPD medium at 30°C for 12 h. As shown in Fig. 1, *EHT1* levels dropped by 59 and 66% in the *Δeht1* and *Δeht1Δeeb1* strains, respectively (Fig.1A), and that of *EEB1* was reduced by 51 and 58% in the *Δeeb1* and *Δeht1Δeeb1* strains, respectively, compared to the wild type cells (Fig. 1B). Therefore, the mutants still retained ~50% of the expression level of target genes. Interestingly, neither *EHT1* nor *EEB1* was significantly upregulated in the *Δeeb1* and *Δeht1* strains, respectively (*p* > 0.05), suggesting that neither gene compensates for the loss-of-function of the other. Therefore, we hypothesized that despite incomplete deletion of *EHT1* and *EEB1*, ethyl ester synthesis can still be downregulated in the relevant mutants, resulting in an altered ester profile.

### Fermentation Characteristics of the Mutant Strains

**Fig. 2:**
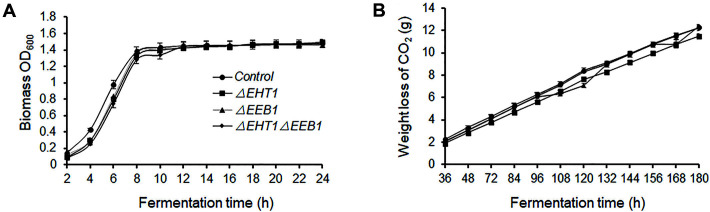
Growth curve (**A**) and CO_2_ loss (**B**) of the indicated strains during fermentation. Values are the mean ± SD of three experiments.

The fermentation ability of the *Δeht1*, *Δeeb1* and *Δeht1Δeeb1* strains was assessed in terms of their growth curves and loss of CO_2_. The respective strains were inoculated into CDG medium, *i.e.*, simulated grape juice medium with a nutrient composition similar to that of grape juice [25], which was supplemented with 100 mg/l yeast available nitrogen (YAN), 200 g/l of an equimolar mixture of glucose and fructose, and the pH was adjusted to 3.5. Fermentation was performed in triplicates over a period of 12 days. As shown in Fig. 2, the lag phase of the three mutants was longer than that of wild-type EC1118, although the growth rate, final cell mass and CO_2_ loss were similar. In addition, there were no significant differences in the contents of residual sugar, alcohol and total acids in the simulated grape juice after 12 days of fermentation by the mutant and control strains (Table S3). Taken together, deletion of one *EHT1* or *EEB1* allele did not significantly affect the fermentation ability of the mutant strains.

### Content of Esters and Higher Alcohols and the Expression Profiles of Relevant Genes During Fermentation

**Fig. 3:**
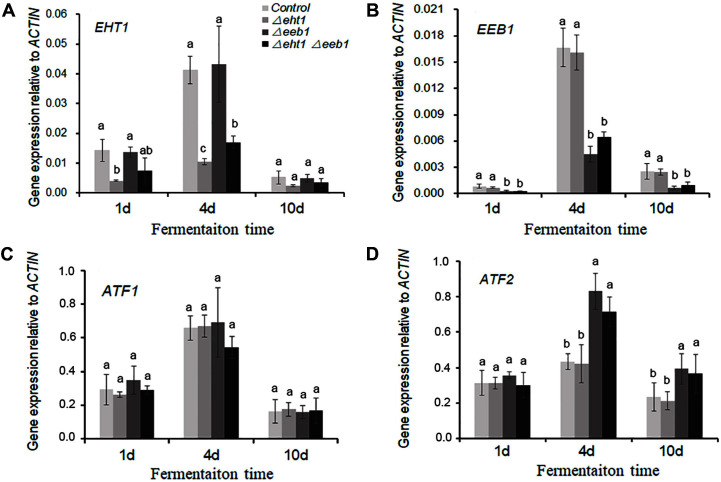
Relative expression levels of *EHT1* (**A**), *EEB1* (**B**), ATF1 (**C**) and *ATF2* (**D**) in the yeast strains on days 1, 4 and 10 of simulated grape juice fermentation. Values are the mean ± SD of three experiments. Different letters (a-c) in the same fermentation period indicate significant differences between strains calculated by the LSD test (*p* < 0.05).

The content of esters and higher alcohols in the CDG medium was also measured on day 12 of fermentation with mutants and control strains (Table 1). In addition, we also analyzed the expression profiles of *EHT1*, *EEB1*, and of *ATF1* and *ATF2*, in the three mutants at different time points during fermentation (Fig. 3). The results are discussed in the following sections.

**Ethyl Esters**. MCFA ethyl ester biosynthesis in *S. cerevisiae* is catalyzed by the *EEB1* and *EHT1*-encoded O-acyltransferases [10]. We observed a decrease in the production of ethyl esters by all three mutants compared to the wild-type strain (Table 1). Furthermore, *EEB1* expression was consistently lower in the *Δeeb1* and *Δeht1Δeeb1* strains compared to that in the control and *Δeht1* strains during fermentation, and decreased in all strains after day 4. Similar trends were seen with *EHT1* as well. However, studies show that *EEB1* plays the dominant role in ethyl ester production [4]. Consistent with this, the amount of ethyl esters was reduced by ~36% in both the *Δeeb1* and *Δeht1Δeeb1* strains, as opposed to only 29% in the *Δeht1* strain (Table 1) compared to the wild-type strain. Nevertheless, the decrease in *EHT1* was slightly more in the *Δeht1* strain compared to that of *EEB1* in the *Δeeb1* strain (Figs. 3A and 3B). In addition, ethyl ester production did not drop further in the double mutant compared to that in the *Δeeb1* strain, which raises the possibility of a limited compensatory effect of other minor alcohol acyltransferases such as MR210w [10] during simultaneous downregulation of *EEB1* and *EHT1*.

Ethyl hexanoate levels were the lowest in the *Δeeb1* strain and showed a 52% reduction compared to those in the wild-type strain (Table 1), which is consistent with a previous finding that the maximum expression level of *EEB1* correlates significantly with ethyl hexanoate concentration (Fig. 3) [13, 26]. Since *EHT1* catalyzes ethyl ester synthesis as well as hydrolysis, some studies on *EHT1*-overexpressing yeast strains proposed a strong negative correlation between *EHT1* expression and the final concentrations of ethyl octanoate and decanoate [24, 26]. In our study, however, *EHT1* expression was negatively correlated to only the final concentration of ethyl decanoate in the mutant strains (Table 1, Fig. 3). In line with the *EHT1* expression level in the *Δeeb1* and *Δeht1* strains, the content of ethyl octanoate was slightly higher in the former (Table 1). This indicates that when *EHT1* is expressed at lower levels, the content of ethyl octanoate may be largely determined by the esterase and not the synthesis activity of *EHT1*.

**Acetate esters**. Acetate esters are an important group of wine esters that are synthesized by alcohol acetyltransferases from acetate and alcohols. In *S. cerevisiae* cells, alcohol acetyltransferases are encoded by the ATF genes [17, 27]. Three acetate esters were detected in this study, including ethyl acetate, isoamyl acetate and phenethyl acetate (Table 1). Compared to the wild-type strain, the content of acetate esters did not change significantly in *Δeht1*, but showed a slight increase in the *Δeeb1* and *Δeht1Δeeb1* strains. We also analyzed the expression levels of ATFs during fermentation, and found that irrespective of the strain, both ATF1 and *ATF2* peaked on day 4 and decreased thereafter. However, *ATF2* expression levels were significantly higher in the *Δeeb1* and *Δeht1Δeeb1* strains compared to that in wild-type and *Δeht1* strains on day 4, whereas ATF1 was expressed at similar levels in all strains at the same time point (Fig. 3C). A previous study showed that the deletion of *ATF2* resulted in only a minor decrease in acetate ester production as opposed to that of ATF1 [19]. Likewise, we found that the upregulation of *ATF2* in the *Δeeb1* and *Δeht1Δeeb1* strains caused only a slight increase in the content of acetate esters (Table 1, Fig. 3D). It is worth noting that *ATF2* expression may be affected by *EEB1* across the different strains grown under the same culture conditions, although the underlying mechanisms are unclear. In contrast, deletion or overexpression of ATFs does not affect ethyl ester synthesis [19]. Altogether, we proposed that in contrast to *EHT1*, regulating *EEB1* expression may modify the profile of ethyl esters and acetate esters more effectively during fermentation.

**Fig. 4:**
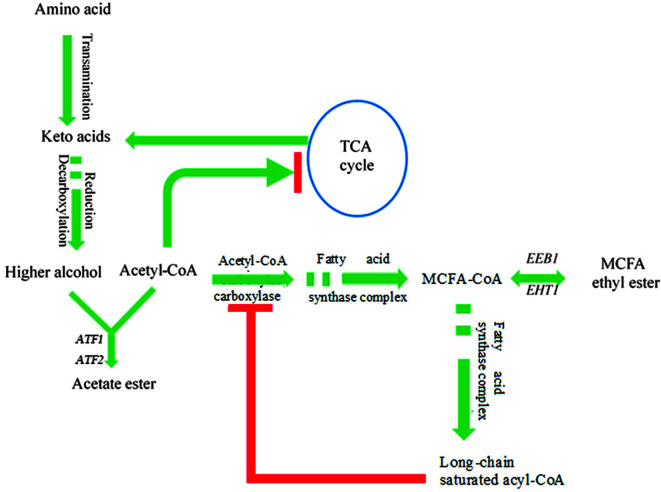
The correlation between acetyl-CoA and ester formation under brewing fermentation conditions (limited amount of oxygen). Red line represents inhibition.

**Higher alcohols**. Higher alcohols are volatile compounds that are mainly derived from amino acid degradation via the Ehrlich pathway during fermentation [14, 28]. They are the precursors of acetate esters, and therefore influence the ester profile of the fermented product [29, 30]. The *Δeeb1* and *Δeht1Δeeb1* strains significantly increased the content of higher alcohols (Table 1), indicating that *EEB1* activity influences their biosynthetic pathway. Given that MCFA ethyl esters are the product of ethanol O-acyltransferase-catalyzed condensation of MCFA acyl-CoA and ethanol, downregulation of *EEB1* results in the accumulation of the precursor MCFAs (Fig. 4). The MCFAs are then fed into the elongation reaction pathway to form complete long-chain fatty acids (FAs) in order to alleviate their toxicity in yeast cells [4, 31]. The FAs can also upregulate the genes encoding for transaminases (**BAT1** and **BAT2**) and decarboxylases (**PDC1**, **PDC5**, **PDC6**, and **ARO10**), which increases the production of higher alcohols via the Ehrlich pathway [14, 24]. On the other hand, during fermentation under limited oxygen levels, the long-chain saturated FAs accumulate and inhibit acetyl-CoA carboxylase, which blocks fatty acid synthesis [32, 33]. In addition, the TCA cycle cannot be (fully) used under anaerobic conditions [4, 34](Fig. 4), which renders all regular acetyl-CoA-consuming pathways inactive and leads to the accumulation of acetyl-CoA. Since *EEB1* downregulation led to an increase in *ATF2* expression level (see previous section), we surmise that the excess amount of acetyl-CoA or/and higher alcohols produced during fermentation by the *Δeeb1* and *Δeht1Δeeb1* strains induce ATF2 to produce more ethyl acetate (Figs. 3C and 3D). In our next study, we will analyze the content of FAs and the expression levels of genes involved in higher alcohol synthesis in the mutant yeast strains during fermentation, and determine the regulatory effect of *ATF2*.

To summarize, deletion of one allele of *EEB1* or/and *EHT1* in industrial yeast EC1118 decreased production of ethyl esters. Furthermore, the partial downregulation of *EEB1* altered the expression level of *ATF2* and affected the acetate ester profile of wine. Although deleting one allele of the target genes might have a weaker effect on the ester profile of wine as opposed to complete deletion, it might be a more feasible strategy for modifying wine aroma. Modest changes in the concentration of these secondary metabolites can cumulatively affect the final sensorial quality of wine [13]. Therefore, sensory test of wine samples should be performed to evaluate the effect of these mutant strains with greater accuracy.

## Supplemental Materials

Supplementary data for this paper are available on-line only at http://jmb.or.kr.

## Figures and Tables

**Table 1:** The concentration of esters and higher alcohols after 12 days of fermentation.

Compound	Concentration (mg/l)			

	Control	*Δeht1*	*Δeeb1*	*Δeht1* *Δeeb1*
Ethyl butanoate	0.56 ± 0.09a	0.54 ± 0.09a	0.35 ±0.02b	0.48 ± 0.04b
Ethyl hexanoate	5.18 ± 0.45a	3.90 ±0.61b	2.48 ± 0.46c	3.07 ±0.51bc
Ethyl octanoate	36.94 ± 0.81a	25.10 ± 2.83b	27.12 ± 1.98b	24.77 ± 2.68b
Ethyl decanoate	21.75 ± 1.30a	16.54 ± 1.65b	11.14 ± 0.98c	12.79 ± 0.65bc
Total ethyl ester	64.64 ± 0.14a (100%)	46.04 ±0.73b (71%)	41.10 ± 2.14c (64%)	41.03 ± 2.99c (63%)
Ethyl acetate	6.56 ± 0.46a	6.36 ± 0.17a	6.44 ± 1.30a	6.68 ± 2.11a
Isoamyl acetate	2.67 ± 0.65b	3.10 ± 0.63ab	4.31 ±1.01a	3.57 ± 0.66ab
Phenyl ethyl acetate	4.44 ± 0.88b	5.36 ± 1.45ab	6.02 ± 0.12ab	6.65 ± 1.31a
Total acetate ester	13.66 ± 0.67b (100%)	14.82± 0.82ab (108%)	16.77 ± 1.91a (123%)	16.90 ± 2.23a (124%)
Isobutanol	8.93 ± 0.41b	6.78 ± 0.38c	10.88 ± 0.98a	10.54 ± 0.04a
Isoamyl alcohol	63.74 ± 1.94b	65.28 ± 6.38b	78.93 ± 1.03a	75.88 ± 3.96a
Phenyl alcohol	44.23 ± 3.81a	43.66 ± 2.14a	48.55 ± 5.95a	49.68 ± 0.18a
Total high alcohols	116.91 ± 4.26b (100%)	115.71 ± 7.84b (99%)	138.35 ± 7.75a (118%)	136.12 ± 4.12a (116%)

Values are the mean ± SD of three experiments. Different letters (a-c) in the same line indicate significant differences between strains calculated by LSD test (*p* < 0.05). Percentages in brackets are relative to control.

## References

[1] Robinson AL, Boss PK, Heymann H, Solomon PS, Trengove RD (2011). Influence of yeast strain, canopy management, and site on the volatile composition and sensory attributes of *Cabernet sauvignon* wines from Western Australia. J. Agric. Food Chem..

[2] Swiegers JH, Bartowsky EJ, Henschke PA, Pretorius IS (2005). Yeast and bacterial modulation of wine aroma and flavour. Aust. J. Grape Wine Res..

[3] Morales ML, Fierro-Risco J, Callejon RM, Paneque P (2017). Monitoring volatile compounds production throughout fermentation by *Saccharomyces* and non-*Saccharomyces* strains using headspace sorptive extraction. J. Food Sci. Technol..

[4] Saerens SMG, Delvaux FR, Verstrepen KJ, Thevelein JM (2010). Production and biological function of volatile esters in *Saccharomyces cerevisiae*. Microb. Biotechnol..

[5] Mason AB, Dufour JP (2000). Alcohol acetyltransferases and the significance of ester synthesis in yeast. Yeast.

[6] Hu K, Jin GJ, Mei WC, Li T, Tao YS (2018). Increase of medium-chain fatty acid ethyl ester content in mixed *H. uvarum*/*S. cerevisiae* fermentation leads to wine fruity aroma enhancement. Food Chem..

[7] Sumby KM, Grbin PR, Jiranek V (2010). Microbial modulation of aromatic esters in wine: Current knowledge and future prospects. Food Chem..

[8] Procopio S, Qian F, Becker T (2011). Function and regulation of yeast genes involved in higher alcohol and ester metabolism during beverage fermentation. Eur. Food Res. Technol..

[9] Bisson LF, Karpel JE (2010). Genetics of yeast impacting wine quality. Ann. Rev. Food Sci. Technol..

[10] Saerens SM, Verstrepen KJ, Van Laere SD, Voet AR, Van Dijck P, Delvaux FR (2006). The *Saccharomyces cerevisiae*
*EHT1* and *EEB1* genes encode novel enzymes with medium-chain fatty acid ethyl ester synthesis and hydrolysis capacity. J. Biol. Chem..

[11] Lilly M, Bauer FF, Lambrechts MG, Swiegers JH, Cozzolino D, Pretorius IS (2006). The effect of increased yeast alcohol acetyltransferase and esterase activity on the flavour profiles of wine and distillates. Yeast.

[12] Zhuang S, Fu J, Powell C, Huang J, Xia Y, Yan R (2015). Production of medium-chain volatile flavour esters in *Pichia pastoris* wholecell biocatalysts with extracellular expression of *Saccharomyces cerevisiae* acyl-CoA:ethanol *O*-acyltransferase Eht_1_ or Eeb_1_. Springer Plus.

[13] Saerens SM, Delvaux F, Verstrepen KJ, Van Dijck P, Thevelein JM, Delvaux FR (2008). Parameters affecting ethyl ester production by *Saccharomyces cerevisiae* during fermentation. Appl. Environ. Microbiol..

[14] Hazelwood LA, Daran JM, van Maris AJ, Pronk JT, Dickinson JR (2008). The Ehrlich pathway for fusel alcohol production: a century of research on *Saccharomyces cerevisiae* metabolism. Appl. Environ. Microbiol..

[15] Eder M, Sanchez I, Brice C, Camarasa C, Legras JL, Dequin S (2018). QTL mapping of volatile compound production in *Saccharomyces cerevisiae* during alcoholic fermentation. BMC Genomics.

[16] Eden A, Van Nedervelde L, Drukker M, Benvenisty N, Debourg A (2001). Involvement of branched-chain amino acid aminotransferases in the production of fusel alcohols during fermentation in yeast. Appl. Microbiol. Biotechnol..

[17] Fujii T, Yoshimoto H, Tamai Y (1996). Acetate ester production by *Saccharomyces cerevisiae* lacking the ATFl gene encoding the alcohol acetyltransferase. J. Ferment. Bioeng..

[18] Holt S, Trindade de Carvalho B, Foulquie-Moreno MR, Thevelein JM (2018). Polygenic analysis in absence of major effector ATF1 unveils novel components in yeast flavor ester biosynthesis. mBio.

[19] Verstrepen KJ, Van Laere SD, Vanderhaegen BM, Derdelinckx G, Dufour JP, Pretorius IS (2003). Expression levels of the yeast alcohol acetyltransferase genes *ATF1*, *Lg-ATF1*, and *ATF2* control the formation of a broad range of volatile esters. Appl. Environ. Microbiol..

[20] Styger G, Prior B, Bauer FF (2011). Wine flavor and aroma. J. Ind. Microbiol. Biotechnol..

[21] Güldener U, Heck S, Fiedler T, Beinhauer J, Hegemann JH (1996). A new efficient gene disruption cassette for repeated use in budding yeast. Nucleic Acids Res..

[22] Marisa ER, Jesús MC, Emilia M, Agustín A (2008). Btn2p is involved in ethanol tolerance and biofilm formation in flor yeast. FEMS Yeast Res..

[23] Livak KJ, Schmittgen TD (2001). Analysis of relative gene expression data using real-time quantitative PCR and the 2^−ΔΔCT^ method. Methods.

[24] Liu PT, Duan CQ, Yan GL (2019). Comparing the effects of different unsaturated fatty acids on fermentation performance of *Saccharomyces cerevisiae* and aroma compounds during red wine fermentation. Molecules.

[25] Henschke PA, Jiranek V, Fleet GH (1993). Yeast: metabolism of nitrogen compounds. Wine microbiology and biotechnology.

[26] Chen Y, Li F, Guo J, Liu G, Guo X, Xiao D (2014). Enhanced ethyl caproate production of Chinese liquor yeast by overexpressing *EHT1* with deleted FAA1. J. Ind. Microbiol. Biotechnol..

[27] Nagasawa N, Bogaki T, Iwamatsu A, Hamachi M, Kumagai C (1998). Cloning and nucleotide sequence of the alcohol acetyltransferase II gene (*ATF2*) from *Saccharomyces cerevisiae* Kyokai no. 7. Biosci. Biotechnol. Biochem..

[28] Calderbank J, Keenan MH, Rose AH (1985). Plasma-membrane phospholipid unsaturation affects expression of the general aminoacid permease in *Saccharomyces cerevisiae* Y185. J. Gen. Microbiol..

[29] Trotter PJ (2001). The genetics of fatty acid metabolism in *Saccharomyces cerevisiae*. Ann. Rev. Nutr..

[30] Duan LL, Shi Y, Jiang R, Yang Q, Wang Y, Liu P (2019). Effects of adding unsaturated fatty acids on fatty acid composition of saccharomyces cerevisiae and major volatile compounds in wine. S. Afr. J. Enol. Vitic..

[31] Wakil SJ, Stoops JK, Joshi VC (1983). Fatty acid synthesis and its regulation. Ann. Rev. Biochem..

[32] Furukawa K, Yamada T, Mizoguchi H, Hara S (2003). Increased ethyl caproate production by inositol limitation in *Saccharomyces cerevisiae*. J. Biosci. Bioeng..

[33] Varela C, Torrea D, Schmidt SA, Ancin-Azpilicueta C, Henschke PA (2012). Effect of oxygen and lipid supplementation on the volatile composition of chemically defined medium and Chardonnay wine fermented with *Saccharomyces cerevisiae*. Food Chem..

[34] Fujii T, Kobayashi O, Yoshimoto H, Furukawa S, Tamai Y (1997). Effect of aeration and unsaturated fatty acids on expression of the *Saccharomyces cerevisiae* alcohol acetyltransferase gene. Appl. Environ. Microbiol..

