# Supplementary material for: Downregulation of EHT1 and EEB1 in Saccharomyces cerevisiae Alters the Ester Profile of Wine during Fermentation
Source: J Microbiol Biotechnol. 2022 Apr 10;32(6):761–7. doi: 10.4014/jmb.2201.01008 (PMC9628906; doi:10.4014/jmb.2201.01008)
Supplement: Supplementary file 1 [file jmb-32-6-761-supple.pdf]

https://doi.org/10.4014/jmb.2201.01008

Table S1. Primers used in this study.

Primer names	Sequence	Products length (bp)
EA1-F	CCTCGAGTATTTCCTCCG	195
EA1-R	GTACGAAGCTTCAGCTGGCGGAACCATTTTCACCGAC	
loxP-KanMX-loxP-F1	GTCGGTGAAAATGGTTCCGCCAGCTGAAGCTTCGTAC	1648
loxP-KanMX-loxP-R1	GAGCTGGCCGCTTTATAATACTCACTATAGGGAGACCGG	
EB1-F	CCGGTCTCCCTATAGTGAGTATTATAAAGCGGCCAGCTC	203
EB1-R	CCTTGGTAGCCCACGAG	
EA2- F	GGTTGCCTACTTAGTTTTCG	210
EA2- R	GTACGAAGCTTCAGCTGGCGAGGAATTTGGCGCTTAG	
loxP-KanMX-loxP-F2	CTAAGCGCCAAATTCCTCGCCAGCTGAAGCTTCGTAC	1648
loxP-KanMX-loxP-R2	CGCATAACAACACACAAGGACTCACTATAGGGAGACCGG	
EB2- F	CCGGTCTCCCTATAGTGAGTCCTTGTGTGTTGTTATGCG	214
EB2- R	GATTGGAAAGCACAGCG	
RT-qPCR-*EHT1*-F	GACGAGAAGGCGACACATC	198
RT-qPCR-*EHT1*-R	CCACTTGAAATCTCCCACTG	
RT-qPCR-*EEB1*-F	TCTGATGGAGGGGTCTGC	157
RT-qPCR-*EEB1*-R	CTAGTTCTCGGGTGAAGACG	
RT-qPCR-ATF1-F	GGACCGAGTTGGCGGCTAAT	108
RT-qPCR-ATF1-R	GTGGATCGAAGACCGACCATCA	
RT-qPCR-ATF2-F	AACACCCTTCATTCAAGCGT	111
RT-qPCR-ATF2-R	CTTGCGTTGCTTGGAATAGC	
ACTIN1-F	CGTCTGGATTGGTGGTTCTA	83
ACTIN1-R	GTGGTGAACGATAGATGGAC	

Table S2. Strains and plasmids used in this study.

Strains and plasmids	Genotype or construct
Strains	
Escherichia coli DH5α	Host of plasmid
Saccharomyces cerevisiae EC1118	Commercial wine yeast strain
Transformants	
Δeht1(*KanMX*)	Diploid yeast strain, *Δeht1*::*loxP-KanMX1-loxP*
Δeeb1(*KanMX*)	Diploid yeast strain, *Δeeb1*::*loxP-KanMX2-loxP*
Δeht1Δeeb1(*KanMX*)	Diploid yeast strain, *Δeht1Δeeb1*::*loxP-KanMX2-loxP*
Δeht1(*KanMX*,pSH65)	Diploid yeast strain, *Δeht1*::*loxP-KanMX1-loxP*::pSH65
Δeeb1(*KanMX*,pSH65)	Diploid yeast strain, *Δeeb1*::*loxP-KanMX2-loxP*::pSH65
Δeht1Δeeb1(*KanMX*,pSH65)	Diploid yeast strain, *Δeht1Δeeb1*(*KanMX*)::*loxP-kanMX2-loxP*::pSH65
Δeht1	Diploid yeast strain, *Δeht1*::*loxP-loxP*
Δeeb1	Diploid yeast strain, *Δeeb1*::*loxP-loxP*
Δeht1 Δeeb1	Diploid yeast strain, *Δeht1*::*loxP-loxP Δeeb1*::*loxP-loxP*
Plasmids	
pUG6	Amp^r, *loxP-KanMX1-loxP*
pSH65	Amp^r, cer, gene knockout vector

Table S3. Basic characters of simulated wine from fermentation with mutant strains.

Strain	Ethanol (% v/v)	Residual sugar (g/L)	Total acids (g/L)
EC1118	11.0±0.19a	3.79±0.12a	5.02±0.12a
Δeht1	10.9±0. 20a	3.68±0.22a	5.12±0.10a
Δeeb1	10.6±0.30a	3.54±0.14a	5.15±0.16a
Δeht1Δeeb1	10.6±0.22a	3.59±0.11a	5.02±0.21a

Values are the mean ± SD of three experiments. Same letter (a) in the same column indicate no significant difference between strains calculated by LSD't-test ($P > 0.05$).
